# Magnesium and Osteoporosis: Current State of Knowledge and Future Research Directions

**DOI:** 10.3390/nu5083022

**Published:** 2013-07-31

**Authors:** Sara Castiglioni, Alessandra Cazzaniga, Walter Albisetti, Jeanette A. M. Maier

**Affiliations:** 1 Department of Biomedical and Clinical Sciences L. Sacco, University of Milan, Via GB Grassi 74, Milan I-20157, Italy; E-Mails: sara.castiglioni@unimi.it (S.C.); alessandra.cazzaniga@unimi.it (A.C.); 2 Department of Biomedical, Surgical and Dental Sciences, University of Milan, Via Commenda 10, Milan I-20157, Italy; E-Mail: walter.albisetti@unimi.it

**Keywords:** osteoporosis, magnesium, osteoblast, osteoclast

## Abstract

A tight control of magnesium homeostasis seems to be crucial for bone health. On the basis of experimental and epidemiological studies, both low and high magnesium have harmful effects on the bones. Magnesium deficiency contributes to osteoporosis directly by acting on crystal formation and on bone cells and indirectly by impacting on the secretion and the activity of parathyroid hormone and by promoting low grade inflammation. Less is known about the mechanisms responsible for the mineralization defects observed when magnesium is elevated. Overall, controlling and maintaining magnesium homeostasis represents a helpful intervention to maintain bone integrity.

## 1. Introduction

Osteoporosis is a multifactorial disease characterized by loss of bone mass due to a marked bone microarchitecture deterioration [1]. Physiologically, bone is constantly remodeled by concerted and coordinated interactions between osteoclasts, the cells primarily involved in bone resorption, and osteoblasts, which ensure bone formation and mineralization. Osteoporosis results from an imbalance between bone deposition and resorption. The consequent decline of bone mass increases the risk of fractures, in particular hip and spine fractures, which are associated with substantial pain and suffering, disability, and even death [1].

Osteoporosis affects millions people worldwide, predominantly postmenopausal women. In the United States low bone mass is a threat for more than 40 million people [2]. In Europe, the prevalence of osteoporosis is expected to affect more than 30 million people by the year 2050 [3]. Frequently associated with aging, osteoporosis is a major health concern since the aging population will double over the next decade with enormous cost burden on the healthcare systems. Osteoporosis therapies are available and fall into two classes, anabolic drugs, which induce bone formation, and anti-resorptive drugs, which retard bone resorption. In addition, modifications of lifestyle, *i.e.*, regular physical activity, no alcohol, no smoke, balanced diet, are highly recommended in patients with osteoporosis [1]. In general, because osteoporosis reflects peak bone mass determined by factors preceding skeletal maturity [4], there is a growing interest in preventive strategies for decreasing the incidence of osteoporosis in future decades. Dietary interventions are among them. Indeed, nutritional factors are of particular importance to bone health and they are modifiable by providing food-based recommendations. A correct diet is particularly important in the young, before skeletal maturity is reached. While calcium and vitamin D have been the master focus of nutritional prevention of osteoporosis, several additional food constituents—such as phytoestrogens, flavonoids, vitamins A, B, C, E, folate—and minerals among which copper, zinc, selenium, iron fluoride and magnesium (Mg), are known to be important [5]. In particular, a significant association has been found between bone density and the intake of Mg, an essential micronutrient with a wide range of metabolic, structural and regulatory functions [6].

## 2. Magnesium and the Bone: Molecular, Biochemical and Cellular Insights

About 60% of total Mg is stored in the bone. One third of skeletal Mg resides on cortical bone either on the surface of hydroxyapatite or in the hydration shell around the crystal [7]. It serves as a reservoir of exchangeable Mg useful to maintain physiological extracellular concentrations of the cation [6]. Bone surface Mg levels are related to serum Mg. Accordingly, surface bone Mg increases with Mg loading, as described in chronic renal disease [8]. The larger fraction of bone Mg is probably deposited as an integral part of the apatite crystal and its release follows the resorption of bone. Apart from a structural role in the crystals, Mg is essential to all living cells, including osteoblasts and osteoclasts. Intracellularly, Mg is vital for numerous physiological functions. First of all, Mg is fundamental for ATP, the main source of energy in the cells. Moreover, Mg is cofactor of hundreds of enzymes involved in lipid, protein and nucleic acid synthesis. Because of its positive charge, Mg stabilizes cell membranes. It also antagonizes calcium [9] and functions as a signal transducer [10]. It is therefore not surprising that alterations of Mg homeostasis impact on cell and tissue functions.

## 3. Low Magnesium and Osteoporosis: Experimental Evidence

In several studies on different species, dietary Mg restriction promotes osteoporosis [11]. Bones of Mg deficient animals are brittle and fragile, microfractures of the trabeculae can be detected and mechanical properties are severely impaired [12]. Consequently, it is not surprising that a Mg deficient diet has a negative effect on the peri-implant cortical bone markedly decreasing tibial cortical thickness [13].

**Figure 1:**
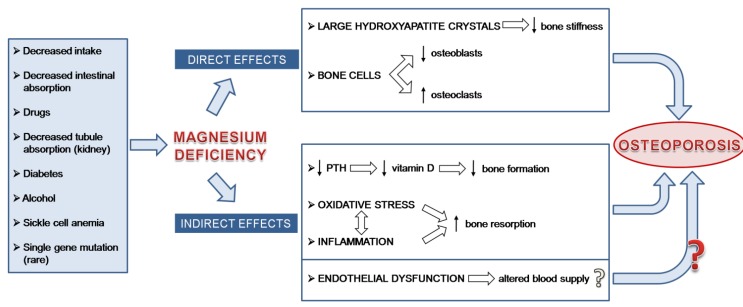
Present knowledge about the mechanisms involved in linking Mg deficiency and osteoporosis. Remarkably, similar events are implicated in experimental models and in humans. Because the vasculature plays an important role in bone remodeling, we also hypothesize that low Mg induced-endothelial dysfunction contributes to the decline of bone mass.

Several direct and indirect mechanisms contribute to the effects of low Mg on bone density (Figure 1). Mg deficiency rapidly leads to hypomagnesemia, which is in part buffered through the mobilization of surface Mg from the bone. In addition, the newly formed crystals of apatite are larger and better structured in Mg deficient animals than controls, and this affects bone stiffness [14]. It should also be recalled that low Mg intake retards cartilage and bone differentiation as well as matrix calcification [15]. In experimental Mg deficiency in rodents, decreased bone formation is partly due to reduced osteoblastic activity [16]. Accordingly, the number of osteoblasts detected by histomorphometry is reduced [17,18] and the levels of two markers of osteoblastic function, namely alkaline phosphatase and osteocalcin, are decreased [14]. Moreover, an increase in the number of osteoclasts has been described [11]. It is noteworthy that these results *in vivo* have been confirmed by *in vitro* studies and some molecular pathways involved have been unraveled. Indeed, low extracellular Mg inhibits osteoblast growth by increasing the release of nitric oxide through the upregulation of inducible nitric oxide synthase [19], while it increases the number of osteoclasts generated from bone marrow precursors [20].

Apart from direct effects on the structure and the cells of the skeleton, Mg deficiency impacts on the bone also indirectly by affecting the homeostasis of the two master regulators of calcium homeostasis, *i.e.*, parathyroid hormone (PTH) and 1,25(OH)_2_-vitamin D thus leading to hypocalcemia. In most species, hypomagnesemia impairs secretion of PTH and renders target organs refractory to PTH. Because PTH signaling implies the increase of cyclic AMP through the activation of adenylate cyclase, which requires Mg as a cofactor, resistance to PTH might be due, in part, to the decreased activity of this enzyme [21]. Reduced secretion of PTH or impaired peripheral response to the hormone lead to low serum concentrations of 1,25(OH)_2_-vitamin D [11,18]. To this purpose it is noteworthy that 25-hydroxycholecalciferol-1-hydroxylase requires Mg [22] and consequently Mg deficiency reduces the activity of the enzyme.

Hypomagnesemia also promotes inflammation [23] and a relation exists between inflammation and bone loss [24]. In Mg deficient rodents, TNFα, IL-1s and IL-6 are increased both in serum and in the bone marrow microenvironment [23]. These cytokines not only amplify osteoclast while inhibiting osteoblast function but also perpetuate inflammation. Besides, substance P is released at high levels in Mg deficiency [18]. In addition to enhancing pro-inflammatory cytokine secretion, substance P released on nerve ending in bone stimulates the activity of the osteoclasts [18].

It is also relevant that Mg restriction promotes oxidative stress, partly as a consequence of inflammation partly because of the reduced anti-oxidant defenses which occur upon Mg restriction [25]. The increased amounts of free radicals potentiate the activity of osteoclasts and depress that of osteoblasts [26].

A last issue that is overlooked in experimental models is about the vasculature in the bone of Mg deficient animals. An adequate blood supply is necessary for bone health. Interestingly, decreased intraosseous blood vessel volume and number seems to be relevant in the development of post-nerve-injury osteoporosis [27] and in old-age associated osteoporosis [28]. Overall, all experimental data from animal studies indicate that reduced dietary intake of Mg is a risk factor for osteoporosis through a constellation of different mechanisms.

## 4. Low Magnesium and Osteoporosis: Studies in Humans

Nutritional monitoring programs have shown an inadequate dietary Mg intake in Europe and North America [29] which leads to subclinical Mg deficiency. This is mainly due to the features of the western diet, rich in processed foods and relatively poor in micronutrients. How can Mg intake be optimized? Since the center of chlorophyll contains Mg, green vegetables are excellent sources of the metal. Also, nuts, seeds, unprocessed grains and some legumes contain large amounts of Mg. However, diet is not the only determinant. For a long time, the existence of differences in Mg handling on a genetic basis has been suspected. Only recently some light has been shed on this issue. In the last decade, rare cases of hypomagnesemia have been linked to hereditary single-gene mutations [30] (Table 1). These uncommon disorders lead to the identification and characterization of some molecular players in Mg homeostasis. These findings fostered studies on the genome and, by single nucleotide polymorphisms, six different genomic regions were individuated that contain variants reproducibly associated with low serum Mg levels [31]. Interestingly, only one of the loci described, namely TRPM6, had a known role in influencing Mg concentrations in humans [32]. These results open new perspectives in our understanding of the complex mechanisms involved in regulating Mg absorption and distribution and should be taken into account when the outcomes of interventional studies are evaluated.

**Table 1:** Inherited disorders leading to hypomagnesemia. The function of the wild type protein is briefly described.

Disease	* Omim^®^ Reference	Gene	Protein
recessive familial hypomagnesemia with hypercalciuria and nephrocalcinosis	248250 248190	CLDN16 CLDN19	Claudin-16 and Claudin-19 Function: tight junction proteins Localization: thick ascending limb of Henle’s loop and convolute distal tubule in the kidney Involved in paracellular epithelial transport
recessive hypomagnesemia with secondary hypolcalcemia	602074	TRPM6	TRPM6 Function: cation channel and α-kinase Localization: intestine and distal convolute tubule in the kidney Involved in transcellular Mg reabsorption in epithelial cells
dominant renal hypomagnesemia	154020	FXYD2	FXYD2 Function: γ-subunit of the Na^+^/K^+^ ATPase Localization: basolateral membrane of proximal and distal tubules in the kidney Involved in transcellular Mg reabsorption
recessive renal hypomagnesemia	131530	EGF	EGF Function: growth factor and magnesiotropic hormone Involved in stimulating magnesium reabsorption in the renal distal convoluted tubule via activation of TRPM6
dominant hypomagnesemia	607803	CNNM2	CNNM2 Function: metal trasporter Localization: ubiquitous; thick ascending limb of Henle’s loop and basolateral membrane of distal tubule in the kidney Involved in renal Mg reabsorption
autosomal dominant myokymia with hypomagnesemia	176260	KCNA1	Kv1.1 Function: voltage-gated K^+^ channel Localization: ubiquitous; distal convolute tubule in the kidney Involved in renal Mg reabsorption

* Omim: Online Mendelian Inheritance in Man.

Because Mg homeostasis is regulated through a complex network of transporters in the intestine and in the kidney, it is not surprising that Mg deficiency is associated with chronic gastrointestinal and renal diseases [32]. It also complicates diabetes mellitus, sickle cell anemia, therapies with some classes of diuretics, antibiotics or anti-neoplastic drugs [33,34]. In addition, it is very common in the elderly and in alcoholists. Some of these conditions share elevated C-reactive protein, a marker of systemic inflammation, as a common denominator and an inverse correlation exists between Mg intake and C-reactive protein [35].

Also in humans Mg deficiency contributes to osteoporosis. Low serum Mg is a co-contributing factor to osteopenia in adults with sickle cell anemia [36]. Moreover, an association between serum Mg and bone density has been reported in pre and post menopausal women [4,37]. Mg intake was positively associated with bone mass density in surviving members of the Framingham study [38]. The same result was obtained in white but not in black males and females (age 70–79), thereby raising the possibility of racial differences in Mg handling [39] which might be explained in the light of the aforementioned genetic variants of genes implicated in Mg homeostasis [31]. In agreement with the aforementioned results, Mg supplementation is beneficial in osteoporotic women [40,41].

Building healthy bone throughout life is a strategy to prevent osteoporosis. To this purpose it is interesting to note that pre-adolescent dietary intake of Mg positively relates to bone mass density in young adulthood as detected by quantitative ultrasound determination of the calcaneus [42] and that Mg supplementation for 12 months has a positive effect on the accrual of bone mass in the hip of peripuberal Caucasian girls [43]. Mg supplementation is therefore important in the periadolescent group, given the suboptimal dietary Mg intake documented in food surveys in western countries. It is also interesting that Mg intake is an independent predictor of bone density in young elite swimmers [44].

The mechanisms explaining the effects of Mg deficiency on the bone in humans are similar to what has been described in experimental models:

(i) low Mg can directly affect the bone by altering the structure of apatite crystals and by acting on bone cells. Indeed, osteoporotic women with demonstrated Mg deficiency have larger and better organized crystals in trabecular bone than controls, while in women undergoing hormone replacement therapy bone Mg is increased and associates with low cristallinity index [45]. We here recall that when crystals are large bones do not bear a normal load.

(ii) Mg deficiency associates with the reduction of the levels of PTH, the induction of end-organ resistance to PTH and the decrease of vitamin D [11,46]. Interestingly, many osteoporotic post-menopausal women who are vitamin D deficient and have low PTH levels are also Mg deficient and Mg supplementation corrects these biochemical abnormalities [47]. Moreover hypomagnesemic diabetic children normalize their levels of 1,25(OH)_2_-vitamin D upon supplementation with Mg [48].

(iii) Mg deficiency associates with low grade inflammation [4,34] and, as mentioned above, inflammatory cytokines stimulate bone remodelling and osteopenia [23].

(iv) Mg deficiency promotes endothelial dysfunction [49] and it is known that endothelial health is important for bone health [50]. On these bases, it is tempting to speculate about the possibility that osteoporosis might be considered a vascular disease of the bone.

(v) Another aspect to consider is the evidence that adults on a western diet develop a low-grade acidosis which is intensified by aging. Recently, the acid load imposed by this diet has been suggested to play a role in the pathophysiology of osteoporosis. Indeed, metabolic acidosis has been shown to lead to calcium loss from bone, to inhibit osteoblast function and stimulate osteoclast activity, and to impair bone mineralization [51]. Accordingly, a neutralizing diet improves bone micro-architecture and bone mineral density [52]. It is therefore feasible that part of the effects of Mg on the skeleton is due to its capability to act as a buffer for the acid produced by the typical western diet [53].

In spite of the evidence showing that Mg is beneficial for the skeleton, warning results were reported in the Women’s Health Initiative Study where it is shown that postmenopausal women with the highest quintile of Mg intake have the highest incidence of wrist fracture [5]. These results are in keeping with some data showing that elevated Mg might have harmful effects on osseous metabolism and parathyroid gland function, leading to mineralization defects. Indeed, high bone Mg inhibits the formation of hydroxyapatite crystals by competing with calcium and by binding to pyrophosphate forming an insoluble salt, not degraded by the enzymes [54]. These events contribute also to osteomalacic renal osteodystrophy and adynamic bone disease [54]. In patients with chronic renal failure or in individuals undergoing dialysis, serum Mg concentrations are frequently elevated and correlate with mineralization defects [54]. Additional intriguing studies were performed on premature infants with osteopenia secondary to MgSO_4_ maternal administration for preterm labor [55,56]. Since Mg is a calcium antagonist [9], it is feasible to propose that high concentrations of Mg alter calcium/Mg ratio, thus leading to dysregulated cell functions. Accordingly, an *in vitro* inhibitory effect of high Mg on osteoblast differentiation and mineralizing activity has been shown [57].

Overall, an optimal range of Mg concentrations might be required to ensure bone homeostasis. More studies are required *in vitro* and *in vivo* about the effects of high Mg concentrations on bone metabolism and structure not only to provide correct nutritional guidelines but also because of the use of Mg as an orthopedic implant material.

## 5. Critical Issues and Future Perspective

Mg has been defined the forgotten electrolyte. Indeed, while a lot of literature is available on calcium, not as much is known about Mg in biomedicine and, specifically, in bone homeostasis. In addition, the measurement of serum Mg is seldom requested in spite of the evidence that hypomagnesemia is very common in industrialized countries. Because Mg (i) interferes with calciotropic hormones and (ii) has been proposed as a natural calcium antagonist, an evaluation of Mg/calcium balance seems to be pivotal in general, and in particular in the case of bone physiology and pathology. To our knowledge, there is only one study showing that the ratio of serum and hair calcium to Mg is a significant indicator of bone mass density [58]. Recent advancement in our knowledge of bone physiology has shown the complexity of the network of molecules involved in maintaining skeletal health. The canonical Wnt pathway is emerging as fundamental for the maintenance of bone homeostasis [59]. Briefly, Wnts are essential in determining the fate of mesenchymal precursors and in regulating osteoblast proliferation, apoptosis, differentiation and function [59]. Accordingly, Wnt antagonist sclerostin is involved in osteoporosis and inflammatory bone loss [60]. No data are available at the moment on this pathway in Mg deficiency. Another hot area of research relates to the use of mesenchymal stem cells (MSC) for regenerative medicine in different fields including orthopaedic surgery. To our knowledge nothing is known about the effects of different concentrations of Mg on MSC survival, growth and differentiation both *in vivo* and *in vitro*, apart from the studies performed with MSC on biodegradable Mg alloys [61]. Also osteocytes have not been studied in relation to Mg. Far from being passive by-standers in the bone, the osteocytes are emerging as mechanotransducers and orchestrators of bone remodelling [62]. Many other challenging questions about Mg and the bone are still unanswered.

## 6. Conclusions

Although the evidence is still fragmentary, most of the experimental and clinical data available in the literature point to Mg as a contributor factor to bone health. Consequently, optimizing Mg intake might represent an effective and low-cost preventive measure against osteoporosis in individuals with documented Mg deficiency, while doubts remain about supplementing the general population with the mineral since too much Mg seems to have detrimental effects on the bone [5,57].
